# Adaptation of Food Craving Inventory to Turkish culture: a validity and reliability study

**DOI:** 10.1186/s40337-022-00667-x

**Published:** 2022-10-06

**Authors:** İrem Çağla Özel, Nurcan Yabancı Ayhan, Özlem Çetiner

**Affiliations:** 1 Department of Nutrition and Dietetics, Faculty of Health Sciences, Atılım University, Ankara, Turkey; 2 Department of Nutrition and Dietetics, Institute of Health Sciences, Ankara University, Ankara, Turkey; 3 Department of Nutrition and Dietetics, Faculty of Health Sciences, Ankara University, Ankara, Turkey

**Keywords:** Food Craving Inventory, Turkish population, Gender, Obesity, Validation

## Abstract

**Introduction:**

The Food Craving Inventory is a 28-item self-report measure of specific food cravings. The inventory consists of 4 factors: high fats, sweets, carbohydrates/starches and fast-food fats.

**Purpose:**

This study was carried out to evaluate the Turkish validity and reliability of the Food Craving Inventory, and to determine the psychometric properties and factor structure of the Turkish version.

**Methods:**

The sample of the study consists of 621 individuals between the ages of 19–50 who voluntarily agree to participate in online survey. Validity and reliability analyses were performed for the Turkish version of Food Craving Inventory (FCI-TR). Confirmatory factor analysis was performed to evaluate the factor structure of the Turkish version of FCI.

**Results:**

Confirmatory factor analysis yielded a four-factor structure as “sweets,” “high-fats,” “carbohydrates/starches” and “fast food fats”. The Cronbach-alpha coefficient for the total score was 0.84; subfactors were calculated as 0.74 for “sweets”, 0.64 for “high-fat foods”, 0.65 for “carbohydrates/starches”, and 0.66 for “fast-food fats”. The scores of the FCI-TR factors and its total score significantly correlated with the sub-factors of Three Factor Eating Questionnaire (TFEQ). A significant correlation was found between body mass index (BMI) and high fats and fast-food fats factor score. Also total and factor scores of the FCI-TR were different between BMI groups.

**Conclusions:**

This study demonstrates that the Turkish version of the FCI is a valid and reliable tool to measure food cravings in the Turkish population. FCI is also correlated with sub-factors of TFEQ.

## Plain English Summary

This study was carried out to adapt the Food Craving Inventory to Turkish. The English version of inventory consists of 4 sub-factors that measure cravings for high-fat foods, carbohydrates/starches, sweets, and fast food fats, and creates a total score. The sample of the study consists of 621 individuals between the ages of 19–50 who voluntarily agree to participate in an online survey. This study revealed that FCI-TR is a valid instrument of specific food cravings in the Turkish adult population. Turkish version of the FCI also consist of 4 sub-factors. Women experienced more food craving for sweets than men. While the most craved food by women was chocolate, men scored significantly higher on bread than women. In addition, a relationship was found between food craving and body weight.

## Introduction

Obesity is a serious public health problem all over the world. This is important because obesity has been associated with increasing rates of obesity-related diseases [1]. Food craving is generally defined as an intense and frequent desire for a particular food or type of foods that is difficult to resist [2]. Food cravings are hedonic responses to food and distinct from hunger. The difference that distinguishes craving from other hunger feelings is that it is food-specific and intensely experienced emotion [3]. People eat not only to satisfy homeostatic hunger, but also to satisfy hedonic hunger. Food craving is a key component of hedonic hunger and hedonic hunger also involves overconsumption. Foods with high carbohydrates, sugars or fats are mostly consumed in the case of hedonic hunger [4].

In the obesogenic environment where there is an increase in availability to palatable and processed foods, a wide range of cues can trigger food cravings [5, 6]. Individuals with overweight and obesity may be more sensitive these food-related cues, leading to more frequent high-caloric food cravings that contain high levels of refined carbohydrates, sugar, and fat [7, 8]. The assessment of specific food cravings is an important objective, because it is a potential contributor for weight gain and obesity. Food cravings increase the likelihood of respective food intake and lead to prospective eating. Food cravings are important predictor of weight gain, contributing to the growing obesity epidemic [5, 9–11]. In individuals without eating disorders, most of food cravings lead to sweets, high-fat foods, and fast-food consumption. Moreover, these type of processed foods have addictive potential and individuals with overweight and obesity oftenly experience these type of food cravings [12–14]. Another explanation for this may be emotional eating due to decreased dopamine releasing. The neurotransmitter dopamine regulates food intake by modulating reward via the mesolimbic pathway in the brain. Several studies have shown that individuals with obesity experience less reward due to a lower dopamine releasing after food intake. This mechanism could play a role in the etiology of overconsumption and consequently obesity [15, 16].

Like many other countries in Europe, Turkey has a high rate of obesity and overweight prevalence. According the Turkey Nutrition and Health Survey, 34.0% of the population was overweight and 31.5% was obese. Women are much more affected by obesity and the prevalence is higher in women than in men in Turkey. In terms of sex, 33.1% of women was obese and 28.5% was overweight, while 23.8% of men was obese and 42.0% was overweight [17]. Some researchers in the literature have reported the sex-related differences in the frequency of craving. Women are more likely than men to experience food cravings. Furthermore, men and women tend to crave different kinds of foods [3, 4, 9, 13, 32]. Considering role of craving in obesity, sex difference in craving may be one of the reason of sex variability in obesity prevalence in Turkey [17].

Various tools have been developed to assess food craving in the recent years [18–20]. Among other tools, Food Craving Inventory is unique because it measures cravings for specific foods in the past month. FCI consists of 4 sub-factors that measure cravings for high-fat foods, carbohydrates/starches, sweets, and fast food fats, and creates a total score [20].

There are differences in the cultural expression of food cravings between countries. For this reason, the creators of the FCI suggest that, according as the cultural context and geographical region, adaptations must be made to the inventory [20]. After the validation of the original inventory, other researchers were able to adapt the inventory as regards both structure and category. Food Craving Inventory has been adapted for many cultures including United Kingdom, Germany, Spain, Brazil, Japan and Iranian [21–25]. The versions of United Kingdom and Germany feature the same four-factor structure [21, 23]. The Spain and Brazil versions of inventory have a three-factor structure [22, 31]. Japan and Iranian versions of the inventory have a five-factor structure [24, 25]. However there is no Turkish version of Food Craving Inventory. Considering the Turkish cuisine, it is obvious that some foods in the original version of the Food Craving Inventory are not appropriate for Turkish culture. Turkey is geographically located at the meeting point of the Asia and Europe continents and the richness of Turkish cuisine is based on variety of products cultivated on the lands of Asia and Europe and numerous cultural interactions in history. Turkish cuisine is famous for its kebabs, sherbet desserts such as baklava, kadayıf and künefe and other specialties. Therefore, in the Turkish version there must be cultural alternatives to some food items in the original inventory [17].

In the light of all this information, the aim of present study was to assess the Turkish validity and reliability of the Food Craving Inventory, and to determine the psychometric properties and factor structure of the Turkish version. Also, we evaluated any sex and body mass index (BMI) differences relating to food cravings.

## Methods

### Study design and procedure

Individuals between the ages of 19–50 were included in the study. Data was collected through a web-based questionnaire using snowball sampling method, delivering a message containing a brief description of our study and the available study link, between November 2021 and January 2022. Individuals with any chronic disease or taking medication related to these diseases were excluded from the study. A total of 621 participants living in Turkey and volunteering to participate in the study were evaluated. Participants completed a demographic questionnaire, FCI and TFEQ.

### Measures

The questionnaire consists of 3 parts. In the first part of the questionnaire, the demographic information (age, gender, educational status, marital status, occupation) and general health information of the participants (whether they have chronic and/or psychological diseases, drugs used for those diseases, etc.) have been questioned. Body weight (kg) and height (cm) information were obtained based on the statements of individuals. Body mass index was calculated by dividing the participant’s weight in kilograms by the square of his/her height in meter (kg/m^2^). In the second part, FCI was applied to the participants. In the third part, Three-Factor Eating Questionnaire was applied to the participants.

### Food Craving Inventory

The FCI is a self-report measure of specific food cravings. The inventory consists of 4 factors or subscales measuring cravings for high fats, carbohydrates/starches, sweets, and fast food fats, and also generates a total score. FCI is a 28-item inventory with eight items on the high fats subscale, eight items on the sweets subscale, eight items on the carbohydrates/starches subscale, and four items on the fast-food fats subscale. The high-fats factor consisted of 8 items that are high in fat. The sample items included fried chicken, sausage, and butter. The sweets factor consisted of 8 items such as chocolate and ice cream. The carbohydrates/starches factor has 8 items such as baked potato and pasta. Fast-food fats subscale has 4 items high in calorie content. These items are pizza, hamburger, French fries, and chips. Regarding cravings, FCI asks “Over the past month, how often have you had cravings?” and then participants rates each food on a 5-point Likert scale ranging from 1 (never) to 5 (always/almost every day) [20].

### Adaptation protocol

Permission for the Turkish adaptation of the Food Craving Inventory, the latest English version of the inventory and the scoring instruction were obtained by e-mail from Leslie Smith, Director of Pennington Biomedical Research Center. In accordance with the translation method, it was translated from English to Turkish and then from Turkish to English by a total of three experts, two of whom have English proficiency. These three translations were evaluated together and the consistency between them was compared and discussed. To better fit the inventory to the target population, some foods in the original version were modified. These foods were evaluated by 5 academics who are professionals in the field of Nutrition and Dietetics. According to academics, some foods were replaced with foods that are more commonly consumed in Turkish culture. By making necessary corrections, an inventory containing 27 foods was obtained.

### Three Factor Eating Questionnaire

Following the procedure in the original study, Three-Factor Eating Questionnaire (TFEQ) was applied to the participants in addition to FCI. TFEQ was adapted to Turkish in 2016 [26]. 21-item questionnaire form measures eating behavior with three sub-factors: uncontrolled eating, emotional eating, and cognitive restraint. Uncontrolled eating evaluates the propensity to lose control over food when hunger is felt and includes nine items. Emotional eating measures the relationship between negative moods such as loneliness, anxiety or low mood, and overeating and includes six items. Cognitive restraint assesses the tendency to control food intake to maintain body weight and consists of six items [27]. In the evaluation of convergent validity, the Spearman correlation coefficient between the composite scores of the two subscales TFEQ and the composite score and factor scores of FCI was calculated. For divergent validity, the “cognitive restraint” subscale of TFEQ was used.

### Statistical analysis

Version 22.0 of SPSS was used for the statistical analysis of the study. The descriptive statistics are expressed as mean ± standard deviations and frequency (%). The normality of the distribution of data was assessed using Shapiro–Wilk and Kolmogorov–Smirnov tests. For sex differences in the scores of FCI-TR items were evaluated with *t* test. The BMI was classified into four group as underweight (< 18.5), normal (≥ 18.5 and < 25), overweight (≥ 25 and < 30), and obese (≥ 30). One-way analysis of variance was used for differences in FCI-TR scores between BMI groups. Pearson correlation was used to assess the relationship between factors of inventory and BMI. In all statistical analyses, significance level was accepted as *p* < 0.05. Confirmatory factor analysis (CFA) was performed to test whether the structure in the original inventory could be achieved with the adapted inventory [28]. CFA was carried out using the R Studio software. As a result, we performed a factor analysis in a sample of 621 women and men.

## Results

Of the 621 participants, 518 were women and 103 were men. About 54% of the participants was college students. The mean age of participants is 26.19 ± 8.98 years and the mean BMI is 22.68 ± 3.96 kg/m^2^. According to BMI values, 10.8% of the participants was underweight, 66.3% was normal weight and 22.9% was overweight-obese. From this 22.9%, women represented 62.7% and men 37.3%. The 12.1% of participants stated that they follow a diet for weight loss.

### Confirmatory factor analysis

The factor structure of the Turkish-adapted FCI was examined using the CFA. According to the CFA analysis, the four-factor structure in the original inventory was confirmed in the Turkish-adapted inventory. CFA yielded four conceptual factors interpreted as sweets, high-fat foods, carbohydrates/starches, and fast-food fats.

In the present study, chi-square (x^2^) good fit index, goodness of fit index (GFI), adjusted goodness of fit index (AGFI), comparative fit index (CFI), Tucker Lewis index (TLI) and root mean square error of approximation (RMSEA) were used as fit indices [29]. When the fit indices are evaluated in general, four-factor structure fits the study data.

### Reliability analysis

The internal consistency of the adapted inventory was evaluated with the Cronbach-alpha reliability coefficients for the whole inventory and it factors separately and the cut-off value was accepted as 0.60 [28]. The Cronbach-alpha coefficient for the total score was 0.84; subfactors were calculated as 0.74 for “sweets”, 0.64 for “high-fat foods”, 0.66 for “fast-food fats”, 0.65 for “carbohydrates/starches”, and 0.70 for “high-fat meats. According to these values, “sweets” factor showed good reliability; the other three factors showed acceptable reliability. Since item-scale correlation values above 0.2 are considered appropriate, items showing an item-scale correlation below 0.2 were eliminated from the analysis [30]. Since the factor structure obtained from the CFA showed acceptable fit, explanatory factor analysis was not performed in this study. Table 1 shows the factor loadings of the items and fit index values.

**Table 1:** Factor loadings of the FCI-TR (n = 621)

Food Items	Sweets	High fats		Carbohydrates/starches		Fast-food fats
Chocolate	0.497					
Cake	0.731					
Cookie	0.682					
Ice cream	0.367					
Spreadable chocolate	0.514					
Sherbet desserts	0.516					
Sweetened beverages	0.398					
Jam/honey	0.415					
Cream		0.307				
Butter		0.355				
Sunflower seeds		0.327				
Sauced wrap/doner		0.348				
Kebab		0.655				
Fried chicken		0.576				
Fried fish		0.488				
Kokorec		0.407				
Bread				0.354		
Rice				0.534		
Mashed potatoes				0.601		
Pasta				0.534		
Bagel				0.495		
Lahmacun				0.419		
Raw meatballs				0.319		
Pizza						0.535
Hamburger						0.674
French Fries						0.675
Chips						0.443
Reliability (Cronbach alpha)	0.74	0.64		0.65		0.66
Fit index	X^2^/sd	GFI	AGFI	CFI	TLI	RMSEA
	4.86	0.94	0.92	0.90	0.91	0.08

### Convergent validity

Following the procedure from the original FCI validation study, TFEQ was used as a measure of convergent validity [27]. We used the validated Turkish version of TFEQ [26]. Convergent validity was evaluated with the correlation values between the four factors of FCI-TR and the three subscales of the TFEQ. The “uncontrolled eating” subscale of the TFEQ was correlated with the four factors and the total score of the FCI-TR (*p* < 0.01). There was a positive correlation between the "emotional eating" subscale of the TFEQ and the total score of FCI-TR (*p* < 0.01). While “emotional eating” and “uncontrolled eating” subscales correlated with food craving, “cognitive restraint” subscale did not correlate with the total score. This findings supports the convergent and divergent validity of the FCI-TR. Table 2 shows the correlation of four factors of FCI-TR with BMI and three subscales of TFEQ. According to these correlations, FCI-TR is suitable for both convergent and divergent validity.

**Table 2:** Correlations between FCI-TR, TFEQ, and BMI

	Cognitive restraint	Emotional eating	Uncontrolled eating	BMI
Sweets	− .082	.223**	.276**	− .031
High fats	− .030	.011	.210**	.141**
Carbohydrates/starches	− .153**	.139**	.266**	.002
Fast-food fats	− .182**	.203**	.334**	− .192**
Total score	− .080	.190**	.358**	− .007

Pearson correlation; *p < 0.05; **p < 0.01; The bold *p* values are considered significant

**Fig. 1:**
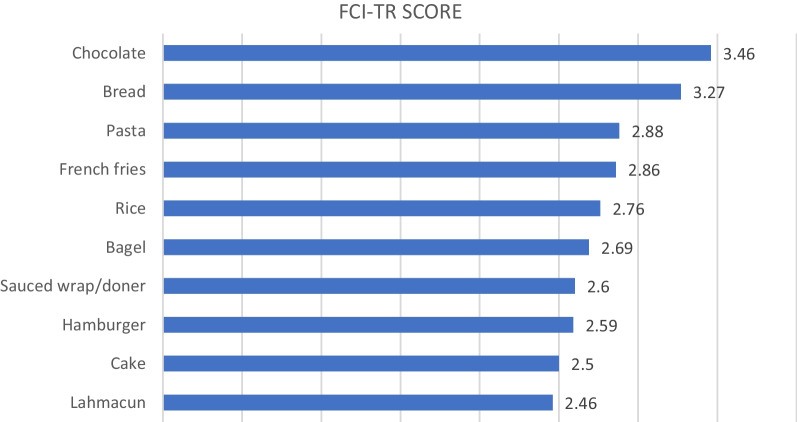
The 10 food items with highest mean scores

Figure 1 shows the 10 foods with the highest average score in FCI-TR. Most craved food was chocolate with a score of 3.46. Bread and pasta were other most craved foods with 3.27 and 2.88, scores, respectively.

Table 3 shows mean scores for each item of the FCI-TR by sex. While the most craved food by women was chocolate, men scored significantly higher on bread than women. The total score of the inventory was not statistically different between women and men (*p* > 0.05).

**Table 3:** FCI-TR item scores by sex

Food Items	Women (n:518)	Men (n:103)	*p*
Pizza	2.34 ± 0.95	2.19 ± 0.87	0.14
Hamburger	2.61 ± 1.06	2.48 ± 1.10	0.25
French fries	2.88 ± 1.06	2.78 ± 0.97	0.40
Sauced wrap/doner	2.57 ± 1.07	2.67 ± 1.04	0.38
Lahmacun	2.43 ± 1.02	2.59 ± 0.95	0.15
Raw meatballs	2.46 ± 1.11	2.23 ± 0.88	0.05
Chips	2.43 ± 1.08	2.04 ± 1.03	< 0.01
Cream	1.53 ± 0.83	1.83 ± 1.05	< 0.01
Butter	2.00 ± 1.17	2.50 ± 1.21	< 0.01
Sunflower seeds	1.96 ± 0.99	1.95 ± 1.03	0.92
Chocolate	3.55 ± 1.10	2.96 ± 1.01	< 0.01
Cake	2.54 ± 1.03	2.30 ± 0.99	< 0.01
Cookie	2.35 ± 1.02	2.05 ± 0.90	< 0.01
Ice cream	2.14 ± 1.06	2.01 ± 0.88	0.27
Spreadable chocolate	2.22 ± 1.07	2.05 ± 1.05	0.15
Sherbet desserts	2.14 ± 1.01	2.60 ± 1.05	< 0.01
Sweetened beverages	2.16 ± 1.10	2.65 ± 1.21	< 0.01
Jam/honey	2.12 ± 1.06	2.40 ± 1.18	< 0.05
Bread	3.21 ± 1.33	3.55 ± 1.16	< 0.01
Rice	2.68 ± 1.10	3.11 ± 0.98	< 0.01
Mashed potatoes	1.94 ± 1.00	1.99 ± 1.00	0.66
Pasta	2.89 ± 1.10	2.81 ± 0.95	0.50
Bagel	2.67 ± 1.04	2.77 ± 0.96	0.38
Fried chicken	2.39 ± 1.07	2.70 ± 0.97	< 0.01
Kebab	2.30 ± 1.04	2.80 ± 1.08	< 0.01
Fried fish	2.10 ± 1.10	2.34 ± 1.05	< 0.05
Kokorec	1.42 ± 0.80	1.94 ± 1.15	< 0.01

Independent-Samples T Test

The FCI-TR factor scores by BMI groups are given in Table 4. In the high fats factor, the mean score of participants with obesity was higher than those of normal weight (*p* < 0.05). In the fast-food fats factor underweight group had the highest score compared to other BMI groups (*p* < 0.05). There was no statistically significant difference between BMI groups for carbohydrates/starches and sweets factor scores (*p* > 0.05).

**Table 4:** FCI-TR factor scores by BMI groups

Factors	Underweight (n:67)	Normal weight (n:412)	Overweight (n:110)	Obese (n:32)	*p*
Sweets	2.58 ± 0.65	2.32 ± 0.57	2.39 ± 0.63	2.42 ± 0.73	0.050
High fats	2.12 ± 0.56^b,c,d^	2.03 ± 0.54^b^	2.24 ± 0.55^c,d^	2.27 ± 0.56^c,d^	0.000
Carbohydrates/starches	2.81 ± 0.74	2.60 ± 0.62	2.62 ± 0.59	2.63 ± 0.51	0.100
Fast-food fats	2.87 ± 0.68^a^	2.56 ± 0.73^b^	2.29 ± 0.66^c^	2.32 ± 0.69^a,b,c^	0.000
Total score	2.55 ± 0.50^a^	2.36 ± 0.46^b^	2.37 ± 0.42^a,b^	2.42 ± 0.44^a,b^	0.020

One-way Anova, Tukey HSD test was used in post-hoc analysis

The same letters in each line indicate a statistically insignificant difference between BMI groups

## Discussion

The general aim of the present study was to analyze the psychometric properties of the Turkish version of the FCI, specifically its factor structure. The reliability analysis showed that internal consistency was good and FCI-TR is psychometrically stable. Furthermore, FCI-TR correlates with the TFEQ, indicating good convergent validity. All these findings show that FCI-TR is a valid instrument to assess food cravings in Turkish population.

There exist marked cross-cultural differences in food cravings. Food Craving Inventory has been adapted for specific countries and respective food cultures, including United Kingdom, Spain, Germany, Japan, Iran and Brazil [18–22, 28]. British English and German versions have the same four-factor structure as the original inventory [21, 23]. Spanish and Brazilian versions have a three-factor structure [22, 31]. Japanese and Persian versions of the FCI revealed a five-factor structure that also included traditional foods [24, 25]. The factor structure of the FCI-TR was examined using the CFA. According to the CFA analysis, the four-factor structure in the original inventory was confirmed in the Turkish-adapted inventory. FCI-TR is a 27-item inventory with eight items on the sweets scale, eight items on the high fats scale, seven items on the carbohydrates/starches scale, and four items on the fast food fats scale. The carbohydrates/starches factor includes bread and rice which are staple foods for Turkish cuisine. This factor also includes Turkish traditional foods such as lahmacun and raw meatballs. The high fats factor includes kebab and kokorec, which are usually consumed as part of traditional Turkish meal.

Food cravings are a commonly experienced phenomenon [9, 32–34]. Chocolate is the most commonly craved food, followed by other high-caloric sweet and savory foods [32–36]. Consistent with this finding, chocolate is the most craved food in FCI-TR (Fig. 1). Also sex difference in chocolate craving is clearer than other foods in studied population. Sex-related differences in cravings for other types of foods have also been reported. There is distinct sex variability in preference for foods that are predominantly sweet compared with savory. Several studies have shown that men are more likely to crave savory and fatty foods (e.g., meat, fish), whereas women are more likely crave for sweet foods (e.g., chocolate, cookie, cake) [32, 37–40]. A sex-related mechanism explaining this may be hormonal or psychological variations related to menstruation and differences in mood-regulating neurotransmitters [9, 33, 41]. Another mechanism may be altered neural reactivity to food cues in reward-related brain areas in women [42, 43].

In this study, women scored significantly higher on sweets such as chocolate, cake, and cookie whereas men scored higher on craving for carbohydrate/starch and high fat foods such as bread, rice, kebab, fried chicken/fish. This findings are compatible with the previous versions of FCI [21, 22, 25]. Also this shows that FCI-TR is successful in showing sex-based differences in food craving.

Food craving is an important phenomenon within the current food environment and often predicts overeating which makes it a reasonable target in the treatment of obesity. Therefore, it is important to measure specific food cravings [5, 12]. Previous studies have found that individuals with overweight and obesity have more frequent food cravings, especially for foods high in fat and carbohydrate, compared to normal-weight people [6, 10, 13]. In the present study, the mean score of participants with obesity in high fats factor was found to be higher than normal weight participants which means that participants with obesity had more frequent craving for high fats foods. Although underweight participants have higher scores in the fast-food fats factor in FCI-DE and FCI-UK, this difference is not statistically significant [21, 23]. In this study, the mean score of the underweight group in the fast-food factor was found to be significantly higher than the obese groups (*p* < 0.05). Similar to this study, one study found that a people with underweight reported much more food cravings compared to people with obesity [44].

There are some strengths of the current study. The biggest strength of this study was major sample size. Sample size is large enough to reproduce the results obtained by the original and other versions of the FCI. Another strength of the study were the use of an online questionnaire which allowed for the participation of volunteers from Turkey. However the study has some limitations. The disparity of female and male participants’ number was one of the limitations. Also BMI values were calculated from self-reported anthropometric measurements. Test–retest analysis was not conducted. Further research should take these limitations into account and assess food intake besides food craving frequency.

## Conclusion

This study revealed that FCI-TR is a valid and reliable instrument of specific food cravings in the Turkish adult population. Also the relationship between the BMI and food cravings have been investigated using the FCI-TR in the studied population. This is the first study in which FCI was adapted to Turkish population. FCI-TR can be used to understand food craving behavior in the Turkish population and may contribute to studies related food cravings. FCI-TR is a potential tool can be used both in research and clinical implications. It is a useful tool for dietitians and researchers who want to develop a plan to help an individual avoid consuming these foods or consume them in moderation in case of cravings. Strategies targeted reducing food cravings may be helpful to reduce public health concerns regarding to obesity and may be beneficial in the treatment of obesity and eating disorders.

## Data Availability

The data sets used and/or analyzed during the current study are available from the corresponding author on reasonable request. The data are not publicly available due to privacy or ethical restrictions.

## Abbreviations

FCI: Food Craving Inventory
CFA: Confirmatory factor analysis
TFEQ: Three Factor Eating Questionnaire
BMI: Body mass index
GFI: Goodness of fit index
AGFI: Adjusted goodness of fit index
CFI: Comparative fit index
TLI: Tucker Lewis index
RMSEA: Root mean square error of approximation
